# Remimazolam Versus Dexmedetomidine for Monitored Anesthesia Care in Patients Undergoing Transfemoral Transcatheter Aortic Valve Implantation: A Randomized Clinical Trial

**DOI:** 10.3390/jcm15187182

**Published:** 2026-09-16

**Authors:** Sung-woo Hyung, Myokyung Choi, Mee Young Chung, Wonjung Hwang

**Affiliations:** 1 Department of Anesthesiology and Pain Medicine, Eunpyeong St. Mary’s Hospital, College of Medicine, The Catholic University of Korea, Seoul 03312, Republic of Korea; sungwh312@nate.com; 2 Department of Anesthesiology and Pain Medicine, Bucheon St. Mary’s Hospital, College of Medicine, The Catholic University of Korea, Bucheon 14647, Republic of Korea; mchoi0529@gmail.com; 3 Department of Anesthesiology and Pain Medicine, Seoul St. Mary’s Hospital, College of Medicine, The Catholic University of Korea, Seoul 06591, Republic of Korea

**Keywords:** cerebral oxygen saturation, conscious sedation, dexmedetomidine, hemodynamics, monitored anesthesia care, remimazolam, transcatheter aortic valve implantation

## Abstract

**Background/Objectives**: Monitored anesthesia care is increasingly used for transfemoral transcatheter aortic valve implantation (tf-TAVI), but the optimal sedative regimen remains uncertain. We compared remimazolam with dexmedetomidine during tf-TAVI. **Methods**: In this single-center randomized trial, 34 patients were assigned 1:1 to remimazolam or dexmedetomidine. Primary outcomes were the number of intraoperative hypotensive episodes, defined as mean arterial pressure <65 mmHg requiring vasopressor treatment, and cumulative phenylephrine and norepinephrine doses. Secondary outcomes included hemodynamic and physiological variables, regional cerebral oxygen saturation (rSO_2_), arterial blood gases, recovery time, surgeon requests for deeper sedation, and procedural outcomes. Longitudinal variables were analyzed using generalized estimating equations with Holm adjustment for time-specific comparisons. **Results**: No significant between-group differences were detected in intraoperative hypotensive episodes or cumulative phenylephrine and norepinephrine doses, and no significant group-by-time interactions were observed for blood pressure. Both left and right rSO_2_ showed significant group-by-time interactions, with higher remimazolam values at selected time points after Holm adjustment. Arterial carbon dioxide tension was higher and arterial pH lower with remimazolam immediately before rapid ventricular pacing and at procedure completion. Recovery was faster with remimazolam (mean ± standard deviation, 13.5 ± 7.2 vs. 32.4 ± 12.5 min; *p* < 0.001), and surgeon requests for deeper sedation were less frequent (11.8% vs. 76.5%; *p* < 0.001). **Conclusions**: Remimazolam was associated with faster recovery and fewer surgeon requests for deeper sedation; however, the trial was not designed or powered to establish equivalence for hypotensive episodes, vasopressor requirements or hemodynamic outcomes.

## 1. Introduction

Severe aortic stenosis is the most common valvular heart disease requiring intervention among older adults and is associated with substantial morbidity and mortality if untreated [1]. Transcatheter aortic valve implantation (TAVI) has become an established treatment for symptomatic severe aortic stenosis in patients with high surgical risk and has expanded to patients with intermediate and low surgical risk owing to advances in valve technology, procedural techniques, and clinical evidence [2,3,4]. General anesthesia has traditionally been used for TAVI because it facilitates transesophageal echocardiographic guidance [5,6]. However, wider adoption of the transfemoral approach and advances in fluoroscopic and echocardiographic imaging have supported the increasing use of monitored anesthesia care (MAC) for transfemoral TAVI [6,7,8,9]. Compared with general anesthesia, MAC has been associated with earlier recovery and mobilization, shorter hospital stays, and avoidance of complications related to endotracheal intubation [7,8,9]. Accordingly, MAC is increasingly used for transfemoral TAVI.

Patients undergoing TAVI are typically older and often have multiple cardiovascular comorbidities, making the maintenance of hemodynamic stability throughout the procedure particularly important [10]. Dexmedetomidine, a highly selective α_2_-adrenergic receptor agonist, is widely used for procedural sedation because it provides cooperative sedation with limited respiratory depression [11]. However, dose-dependent bradycardia and hypotension, as well as transient hypertension during loading, may occur and necessitate vasopressor therapy and other hemodynamic support [12,13,14].

Remimazolam is an ultrashort-acting benzodiazepine that is rapidly metabolized by tissue esterases to an inactive metabolite, resulting in rapid onset, predictable recovery, and limited accumulation [15,16]. Recent studies have shown that remimazolam can provide effective procedural sedation with relatively stable hemodynamics and limited respiratory depression across various procedural settings [17,18,19,20,21]. Comparative studies have further suggested that remimazolam may be associated with a lower incidence of bradycardia than dexmedetomidine, whereas differences in hypotension have been less consistent [22,23,24,25].

Despite these findings, direct evidence comparing remimazolam and dexmedetomidine during transfemoral TAVI under MAC remains limited [20,26]. Detailed comparative characterization of their effects on intraoperative hemodynamics, cerebral oxygenation, vasopressor requirements, recovery, and procedural outcomes remains lacking. Given the increasing use of MAC for transfemoral TAVI, clarifying the relative clinical and physiological effects of these sedatives is important.

To address this gap, this prospective randomized trial aimed to compare remimazolam- and dexmedetomidine-based MAC in patients undergoing transfemoral TAVI. The primary objective was to compare the number of intraoperative hypotensive episodes and cumulative phenylephrine and norepinephrine doses between the two sedation strategies. Secondary objectives were to explore differences in recovery, procedural outcomes, and selected physiological and safety-related measures.

## 2. Materials and Methods

### 2.1. Study Design

This single-center, prospective, randomized clinical trial was conducted at Eunpyeong St. Mary’s Hospital, The Catholic University of Korea. Patients undergoing transfemoral TAVI under MAC were randomly assigned in a 1:1 ratio to either the remimazolam or dexmedetomidine group. The study was conducted as a parallel-group randomized comparison and was not designed within a formal superiority, noninferiority, or equivalence framework. The randomization sequence was generated by the first author using simple randomization without blocking or stratification. The sequence was stored in a computer file and accessed immediately before assignment. The same investigator generated the sequence, enrolled participants, and assigned interventions and therefore had access to the allocation sequence; no independent allocation-concealment mechanism was used. Participants and surgeons were blinded to group allocation and were not informed of the assigned treatment. The anesthesiologists responsible for administering the study drugs were not blinded because the two sedatives required different administration protocols. Outcome assessors, including those performing postoperative clinical assessments, and data analysts were also not blinded to group allocation. No additional physical masking of the study drugs was used. From March 2024 to March 2025, 34 patients were enrolled, with 17 patients allocated to each group.

This study was conducted in accordance with the Declaration of Helsinki and the principles of Good Clinical Practice. Ethical approval was obtained from the Institutional Review Board of Eunpyeong St. Mary’s Hospital, The Catholic University of Korea (IRB No. PC23MISI0091; approval date: 15 June 2023). The trial was prospectively registered with the Clinical Research Information Service (CRIS; KCT0008753; registration date: 10 August 2023) before patient enrollment. Written informed consent was obtained from all participants before enrollment. Patients and members of the public were not involved in the design, conduct, reporting, or dissemination planning of this trial.

### 2.2. Participants

Patients were eligible for inclusion if they were aged ≥ 65 years, scheduled to undergo tf-TAVI under MAC at Eunpyeong St. Mary’s Hospital, and capable of understanding the study and providing voluntary informed consent. The exclusion criteria were as follows: (1) planned tf-TAVI under general anesthesia; (2) age ≥ 90 years; (3) left ventricular ejection fraction <30%; (4) preoperative mechanical circulatory support, including extracorporeal membrane oxygenation; (5) recent myocardial infarction; (6) known hypersensitivity to dexmedetomidine, opioids, or benzodiazepines; (7) contraindications to dexmedetomidine, remifentanil, or remimazolam; (8) end-stage renal disease requiring dialysis; (9) Child–Pugh class C cirrhosis; (10) preoperative delirium; and (11) previous or current use of antidepressants, antipsychotics, benzodiazepines, or other psychotropic medications that could potentially affect sedation requirements or perioperative neurocognitive responses.

### 2.3. Anesthetic Management and Hemodynamic Monitoring

Upon arrival in the operating room, standard monitoring included continuous electrocardiography, pulse oximetry, and noninvasive blood pressure measurement. Noninvasive blood pressure and peripheral oxygen saturation were recorded at 5-min intervals throughout the procedure.

A Masimo Rainbow^®^ sensor (Masimo Corp., Irvine, CA, USA) was applied to a finger not used for pulse oximetry to continuously measure the pleth variability index (PVI), perfusion index (Pi), noninvasive hemoglobin concentration (SpHb), and oxygen reserve index (ORi). Sedation depth was continuously monitored using the Patient State Index (PSi), a processed electroencephalographic index derived from frontal EEG signals obtained using the SedLine^®^ system (Masimo Corp., Irvine, CA, USA). Bilateral regional cerebral oxygen saturation (rSO_2_) was continuously monitored using near-infrared spectroscopy. Before initiation of sedation, an arterial catheter was inserted under local anesthesia to enable continuous invasive blood pressure monitoring because rapid ventricular pacing and valve deployment during TAVI may cause abrupt hemodynamic changes. The left radial artery was preferentially used, whereas the left dorsalis pedis artery was used when radial cannulation was unsuccessful. Serial arterial blood gas analyses were performed during the procedure. Advanced hemodynamic monitoring was performed using the ProAQT^®^ system (PULSION Medical Systems SE, Feldkirchen, Germany), which derives continuous hemodynamic variables from arterial pulse-contour analysis. The system was initialized by entering the required patient demographic information, and initial hemodynamic values were automatically estimated from the arterial pressure waveform. No transpulmonary thermodilution or external cardiac-output calibration was performed, and the system was not recalibrated after valve deployment. The system continuously provided measurements of cardiac index (CI), stroke volume index (SVI), stroke volume variation (SVV), pulse pressure variation (PPV), systemic vascular resistance index (SVRI), cardiac power index (CPI), and maximum rate of arterial pressure rise derived from pulse-contour analysis (dP/dtmax). After all monitors had been placed, the patients were allowed to stabilize for approximately 5 min before baseline measurements were obtained. High-flow nasal oxygen was initiated before sedation using the Optiflow system with an MR810ARU heated humidifier (Fisher & Paykel Healthcare, Auckland, New Zealand), connected directly to the wall oxygen supply at 20 L/min. An air–oxygen blender was not used, and the source gas consisted of 100% oxygen. Continuous end-tidal carbon dioxide (EtCO_2_) monitoring was maintained throughout sedation as part of routine respiratory surveillance.

Because transfemoral TAVI is frequently accompanied by hemodynamic instability, phenylephrine (100 μg/mL) and norepinephrine were prepared before sedation. Norepinephrine was prepared at a patient-specific concentration by diluting 0.03 mg/kg of norepinephrine to a total volume of 50 mL (equivalent to 0.6 μg/kg/mL). Hypotension was defined as a mean arterial pressure (MAP) < 65 mmHg. Initial treatment consisted of a 100 μg intravenous bolus of phenylephrine. Continuous phenylephrine and/or norepinephrine infusions were initiated when hypotension persisted despite repeated bolus administration. Persistent hypotension despite continuous vasopressor support was treated with 100 μg of intravenous epinephrine as rescue therapy. Vasopressor infusions were gradually tapered once MAP remained ≥65 mmHg.

In the remimazolam group, sedation was induced with a 2.5-mg intravenous bolus of remimazolam, followed by a continuous infusion at 0.2 mg/kg/h that was titrated to the target sedation level, up to a maximum of 0.5 mg/kg/h. In the dexmedetomidine group, a loading dose of 1 μg/kg was administered over 10 min, followed by a continuous infusion at 0.2 μg/kg/h that was titrated up to 0.7 μg/kg/h. The target sedation level was a Ramsay Sedation Scale (RSS) score of 5–6. All patients received remifentanil by target-controlled infusion for procedural analgesia, with the target plasma concentration maintained between 0.5 and 2.0 ng/mL. Upon completion of the procedure, the assigned sedative and remifentanil were discontinued, and all patients were transferred to the cardiac intensive care unit with supplemental oxygen delivered through a facemask at a rate of 5 L/min.

### 2.4. Data Collection

Hemodynamic and physiological variables were recorded at the following nine predefined time points: T1, baseline after arterial catheter placement and hemodynamic stabilization; T2, achievement of the target sedation level; T3, initiation of the procedure; T4, 10 min after procedural initiation; T5, immediately before rapid ventricular pacing; T6, 5 min after valve deployment; T7, 10 min after valve deployment; T8, completion of the procedure; and T9, arrival in the cardiac intensive care unit.

Heart rate (HR), noninvasive mean blood pressure (MBP), and peripheral oxygen saturation (SpO_2_) were recorded at all nine time points. Invasive mean arterial pressure (MAP), ProAQT-derived hemodynamic variables (CI, SVI, SVV, PPV, SVRI, CPI, and dP/dtmax), bilateral rSO_2_, PVI, Pi, ORi, SpHb, and PSi were recorded from T1 through T8 and were not obtained at T9 after transfer to the cardiac intensive care unit. In this study, MAP refers to invasive arterial-line mean pressure, whereas MBP refers to the mean pressure obtained by noninvasive cuff measurement.

Serial arterial blood gas analyses were performed at three predefined time points: A1, immediately after arterial catheter placement and before initiation of sedation; A2, immediately before rapid ventricular pacing; and A3, at completion of the procedure.

The cumulative doses of phenylephrine, norepinephrine, and epinephrine administered from the initiation of sedation to completion of the procedure were also recorded.

### 2.5. Outcomes

The primary outcomes were the number of intraoperative hypotensive episodes and cumulative doses of phenylephrine and norepinephrine. A hypotensive episode was defined as MAP < 65 mmHg requiring vasopressor treatment. During ongoing vasopressor therapy, persistent or recurrent MAP < 65 mmHg requiring an additional vasopressor bolus or an increase in infusion rate was counted as an additional episode. Transient hypotension associated with rapid ventricular pacing was not counted unless MAP remained <65 mmHg 3 min after valve deployment and required vasopressor treatment. Cumulative phenylephrine and norepinephrine doses administered from initiation of sedation until completion of the procedure were recorded separately in micrograms. No dose conversion between phenylephrine and norepinephrine was performed.

The secondary outcomes comprised the following domains: serial hemodynamic variables, procedural variables, cerebral oxygenation and peripheral physiological monitoring variables, sedation depth, adverse clinical outcomes, and serial arterial blood gas variables. Serial hemodynamic variables included heart rate, MAP, MBP, CI, SVI, SVV, PPV, SVRI, CPI, and dP/dtmax. Procedural variables included recovery time, cumulative remifentanil dose, surgeon requests for deeper sedation, pre-balloon valvuloplasty, carotid shielding, and valve recapture. Recovery time was defined as the interval from discontinuation of the assigned study sedative to the first achievement of an RSS score of 2. A surgeon request for deeper sedation was recorded when the surgeon judged the patient to be insufficiently sedated, including apparent wakefulness, or when patient movement prompted the surgeon to request a deeper level of sedation. Cerebral oxygenation and peripheral physiological monitoring variables included bilateral rSO_2_, PVI, Pi, ORi, and SpHb, whereas sedation depth was assessed using PSi. Adverse clinical outcomes included atrioventricular block and clinically identified postoperative delirium. After recovery of consciousness, postoperative mental status was assessed clinically by evaluating orientation to person, place, time, and the recent procedure. Brief orientation assessments have been used in postoperative cognitive recovery assessment and as components of validated delirium-screening instruments in the post-anesthesia setting [27,28]. For the purposes of this study, patients who were unable to appropriately identify their name, current location, current time, or the procedure they had undergone were classified as having clinically identified postoperative delirium based on this orientation assessment. This assessment represented a clinical orientation-based screen rather than a validated delirium diagnostic instrument. Serial arterial blood gas variables, including arterial pH, arterial carbon dioxide tension (PaCO_2_), and arterial oxygen tension (PaO_2_), were evaluated to assess perioperative respiratory and metabolic changes.

### 2.6. Sample Size Calculation

The sample size calculation was based on a pilot study conducted at our institution comparing dexmedetomidine and remimazolam during MAC for transfemoral TAVI. In that study, postoperative MAP was significantly lower in the dexmedetomidine group than in the remimazolam group (mean ± standard deviation [SD], 65.0 ± 3.74 mmHg vs. 71.4 ± 7.09 mmHg). Based on these data, Cohen’s *d* was 1.13.

The sample size was calculated using G*Power version 3.1.9.7 (Heinrich Heine University, Düsseldorf, Germany) for a two-sided independent-samples *t*-test with an α level of 0.05 and 80% statistical power. The minimum required sample size was 14 patients per group. Assuming a dropout rate of 20%, the target sample size was increased to 17 patients per group, resulting in a total enrollment target of 34 patients.

Because the sample-size calculation was based on an anticipated between-group difference in postoperative MAP, the study was not specifically powered to detect differences in the number of hypotensive episodes, cumulative phenylephrine and norepinephrine doses, or the multiple secondary physiological outcomes. No interim analyses were performed, and no formal stopping rules were prespecified.

### 2.7. Statistical Analysis

Statistical analyses of non-longitudinal variables were performed using SPSS software version 18.0 (IBM Corp., Armonk, NY, USA). Continuous variables were summarized as the mean ± SD, whereas categorical variables were summarized as the number and percentage [*n* (%)]. Continuous variables were compared using Welch’s *t*-test or the Mann–Whitney *U* test, as appropriate. Categorical variables were compared using Fisher’s exact test, as appropriate.

Longitudinal hemodynamic, physiological, cerebral oxygenation, sedation depth, and arterial blood gas variables were analyzed using generalized estimating equations (GEE) with a Gaussian distribution and identity link. An exchangeable working correlation structure was specified to account for within-patient correlation, and robust sandwich standard errors were used. Each model included treatment group, time as a categorical factor, and the group-by-time interaction. The overall difference in temporal profiles between treatment groups was assessed using a joint Wald test of the group-by-time interaction terms.

Time-specific between-group contrasts were estimated from model-based marginal means and are reported as the estimated mean difference (remimazolam minus dexmedetomidine) with pointwise 95% confidence intervals. To account for multiple comparisons, *p*-values for time-specific contrasts were adjusted using the Holm method separately within each outcome. For bilateral rSO_2_, the two hemisphere-specific group-by-time interaction tests were adjusted using the Holm method across the two tests, and time-specific contrasts were adjusted across all 16 comparisons (eight time points × two hemispheres). Confidence intervals were not multiplicity-adjusted.

GEE analyses were performed using all available observations without imputation. Measurements unavailable by design were not considered missing observations. No important changes to the prespecified trial methods or outcomes were made after trial commencement. For SVI, SVV, PPV, and dP/dtmax, which had incomplete longitudinal data, additional complete-case sensitivity analyses were performed post hoc to assess the robustness of the findings. All tests were two-sided, and *p* < 0.05 was considered statistically significant; for time-specific contrasts, statistical significance was determined using Holm-adjusted *p*-values. GEE models and estimated marginal mean analyses were performed using the geepack and emmeans packages in R version 4.5.1 (R Foundation for Statistical Computing, Vienna, Austria).

## 3. Results

### 3.1. Baseline and Procedural Characteristics

**Figure 1:**
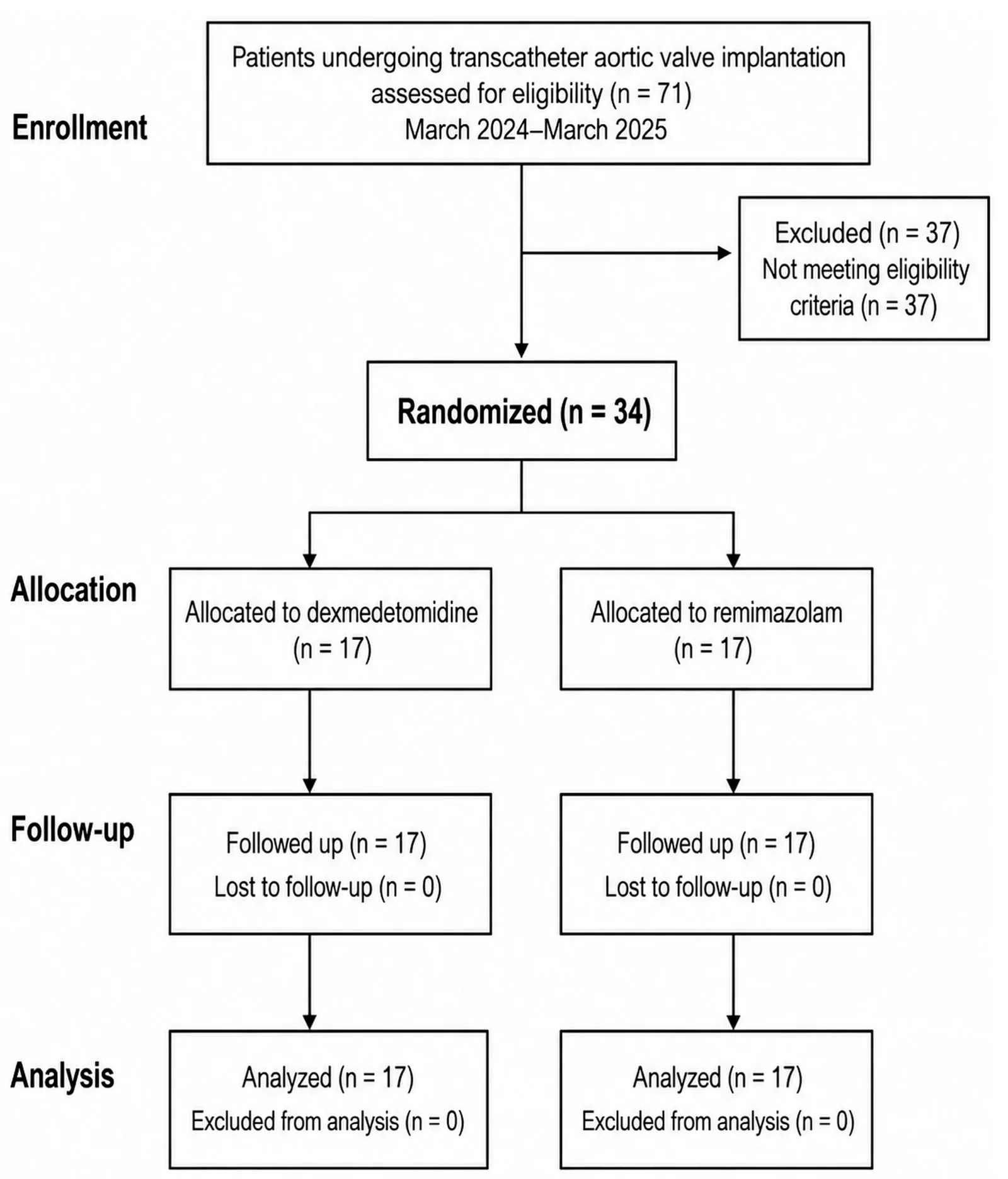
CONSORT flow diagram of participant enrollment, allocation, follow-up, and analysis.

A total of 34 patients were enrolled and randomly assigned to either the dexmedetomidine group (*n* = 17) or the remimazolam group (*n* = 17). All randomized participants received the allocated intervention, and no crossover occurred. All participants completed the study and were analyzed in the group to which they were randomized, with complete data available for the primary outcomes. Follow-up for study outcomes continued through the immediate postoperative clinical assessment after recovery of consciousness. The trial ended as planned after completion of the target enrollment (Figure 1).

Baseline demographic and clinical characteristics, together with procedural and anesthetic characteristics, are summarized in Table 1. The mean total dose of the assigned study sedative was 172.2 ± 40.3 μg for dexmedetomidine and 28.6 ± 9.7 mg for remimazolam.

### 3.2. Primary and Perioperative Outcomes

The primary and perioperative outcomes are summarized in Table 2. For the primary outcomes, no statistically significant between-group differences were detected in the number of intraoperative hypotensive episodes (3.1 ± 3.7 vs. 2.1 ± 2.9; *p* = 0.477), cumulative phenylephrine dose (263.8 ± 376.1 vs. 161.2 ± 208.3 μg; *p* = 0.512), or cumulative norepinephrine dose (333.4 ± 663.7 vs. 302.9 ± 695.7 μg; *p* = 0.762). The proportion of patients experiencing at least one hypotensive episode was 70.6% (12/17) in the dexmedetomidine group and 52.9% (9/17) in the remimazolam group (*p* = 0.481).

Among the other perioperative outcomes, recovery time was shorter in the remimazolam group than in the dexmedetomidine group (13.5 ± 7.2 vs. 32.4 ± 12.5 min; *p* < 0.001), and surgeon requests for deeper sedation were less frequent with remimazolam (2/17 [11.8%] vs. 13/17 [76.5%]; *p* < 0.001). No statistically significant between-group difference was detected in cumulative remifentanil dose (245.0 ± 85.7 vs. 245.8 ± 54.6 μg; *p* = 0.992). No statistically significant between-group differences were detected in the procedural or postprocedural outcomes, including valve recapture, pre-balloon valvuloplasty, carotid shielding, atrioventricular block, and clinically identified postoperative delirium.

### 3.3. Serial Hemodynamic Changes

**Figure 2:**
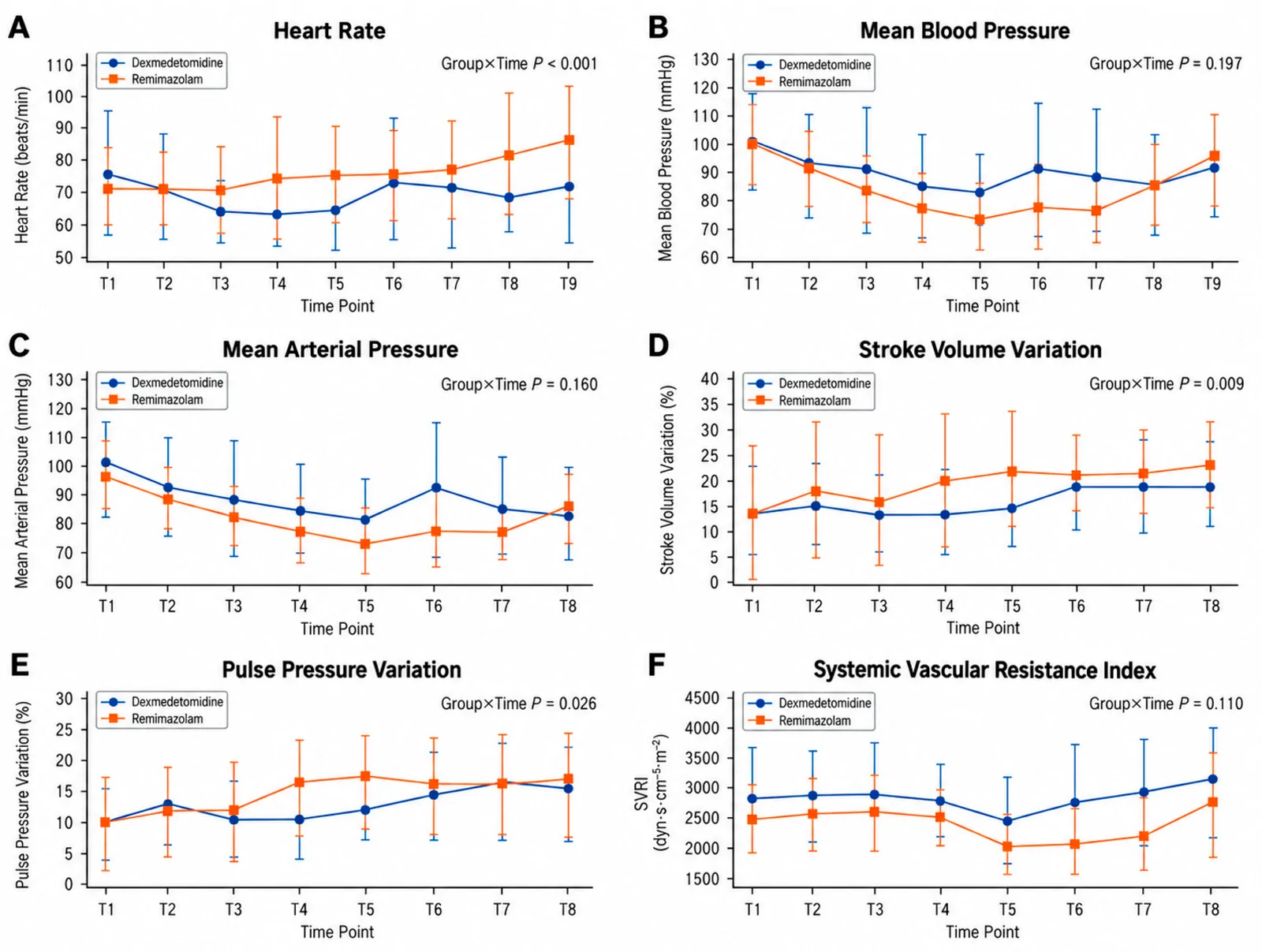
Serial changes in hemodynamic variables during transfemoral transcatheter aortic valve implantation under monitored anesthesia care. (**A**) Heart rate, (**B**) mean blood pressure, (**C**) invasive mean arterial pressure, (**D**) stroke volume variation, (**E**) pulse pressure variation, and (**F**) systemic vascular resistance index. Data are presented as mean ± SD. Longitudinal temporal profiles were analyzed using generalized estimating equations including treatment group, time, and the group-by-time interaction. *p* values displayed in each panel represent the overall group-by-time interaction. Time-specific between-group contrasts were adjusted for multiple comparisons using the Holm method.

Serial changes in heart rate and hemodynamic variables are shown in Figure 2. GEE analysis demonstrated a significant group-by-time interaction for heart rate (*p* < 0.001), indicating different temporal patterns between the two treatment groups. However, none of the time-specific between-group contrasts for heart rate remained statistically significant after Holm adjustment.

Significant group-by-time interactions were also observed for SVV (*p* = 0.009), PPV (*p* = 0.026), and SVI (*p* < 0.001). However, none of the time-specific between-group contrasts for these variables remained statistically significant after Holm adjustment. In contrast, no significant group-by-time interactions were observed for MBP (*p* = 0.197), invasive MAP (*p* = 0.160), or SVRI (*p* = 0.110). Post hoc complete-case sensitivity analyses for SVI, SVV, and PPV yielded results consistent with the primary analyses.

Overall group-by-time interaction tests and detailed time-specific estimated mean differences with 95% confidence intervals and Holm-adjusted *p*-values are provided in Supplementary Tables S1 and S2, respectively.

### 3.4. Sedation Depth, Cerebral Oxygenation, and Peripheral Physiological Variables

**Figure 3:**
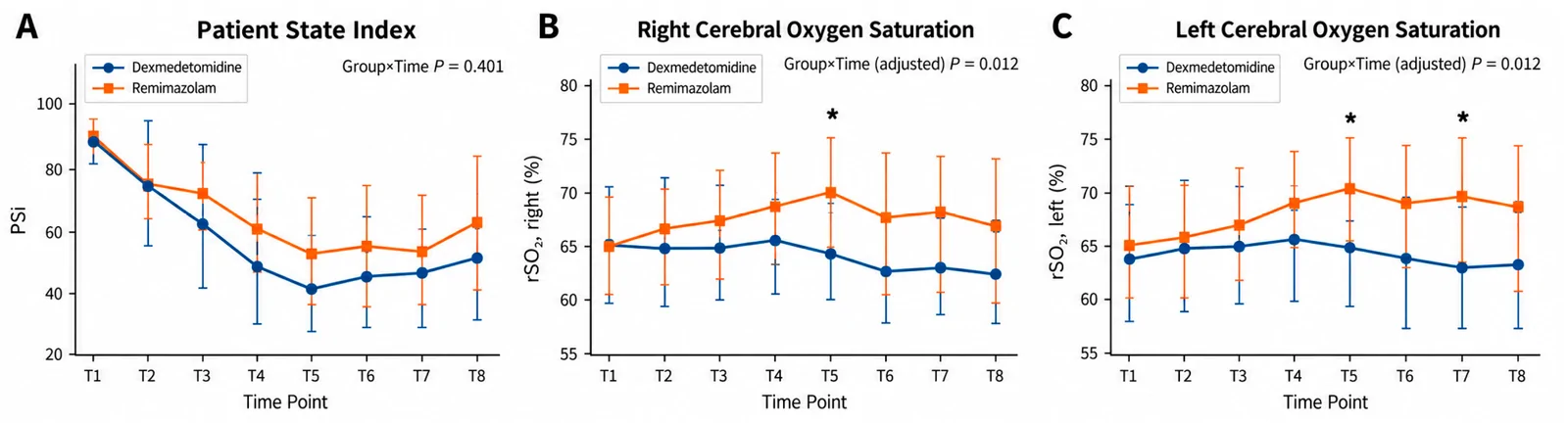
Serial changes in sedation depth and cerebral oxygenation during transfemoral transcatheter aortic valve implantation under monitored anesthesia care. (**A**) Patient State Index (PSi), (**B**) right regional cerebral oxygen saturation (rSO_2_), and (**C**) left regional cerebral oxygen saturation. Data are presented as mean ± SD. Longitudinal temporal profiles were analyzed using generalized estimating equations including treatment group, time, and group-by-time interaction. For bilateral rSO_2_, the hemisphere-specific group-by-time interaction tests were adjusted using the Holm method across the two hemispheres, and time-specific between-group contrasts were adjusted across all 16 comparisons (eight time points × two hemispheres). *p*-values displayed within panels represent the overall group-by-time interaction; for rSO_2_ panels, these are Holm-adjusted values. Asterisks indicate time-specific between-group differences that remained significant after Holm adjustment (adjusted *p* < 0.05).

Serial changes in sedation depth and bilateral cerebral oxygenation are shown in Figure 3. GEE analysis showed no significant group-by-time interaction for PSi (*p* = 0.401), and no time-specific between-group contrast remained statistically significant after Holm adjustment.

Significant group-by-time interactions were observed for both right and left rSO_2_. After Holm adjustment across the two hemisphere-specific interaction tests, both interactions remained significant (adjusted *p* = 0.012 for each hemisphere). In the multiplicity-adjusted time-specific analyses across both hemispheres, right-sided rSO_2_ was higher in the remimazolam group at T5 (estimated mean difference, 5.59 percentage points; 95% confidence interval, 2.22–8.95; Holm-adjusted *p* = 0.016). Left-sided rSO_2_ was higher in the remimazolam group at T5 (estimated mean difference, 6.00 percentage points; 95% confidence interval, 2.40–9.60; Holm-adjusted *p* = 0.016) and T7 (estimated mean difference, 7.59 percentage points; 95% confidence interval, 3.29–11.89; Holm-adjusted *p* = 0.009).

Significant group-by-time interactions were also observed for PVI (*p* = 0.002), ORi (*p* = 0.011), and SpHb (*p* = 0.003). However, none of the time-specific between-group contrasts for these variables remained statistically significant after Holm adjustment. Global interaction tests and detailed time-specific contrasts are provided in Supplementary Tables S1 and S2.

No decrease in rSO_2_ prompted clinical intervention in either group.

### 3.5. Cardiovascular Performance and Peripheral Perfusion

**Figure 4:**
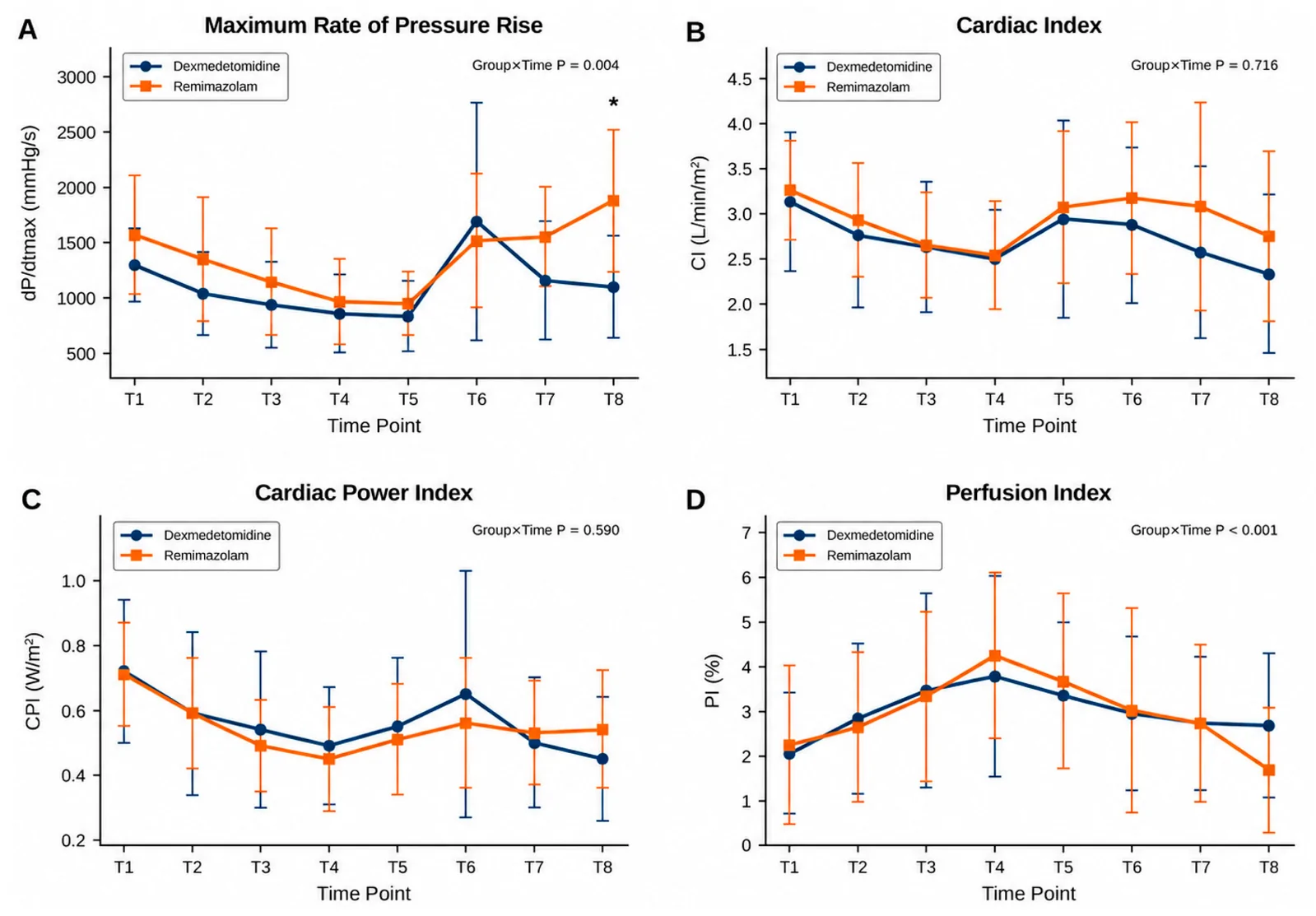
Serial changes in cardiovascular performance and peripheral perfusion during transfemoral transcatheter aortic valve implantation under monitored anesthesia care. (**A**) Pulse-contour-derived maximum rate of arterial pressure rise (dP/dtmax), (**B**) cardiac index (CI), (**C**) cardiac power index (CPI), and (**D**) perfusion index (Pi). Data are presented as mean ± SD. Longitudinal temporal profiles were analyzed using generalized estimating equations including treatment group, time, and the group-by-time interaction. *p*-values displayed in each panel represent the overall group-by-time interaction. Time-specific between-group contrasts were adjusted for multiple comparisons within each outcome using the Holm method. An asterisk indicates a time-specific between-group difference with Holm-adjusted *p* < 0.05.

Selected serial changes in cardiovascular performance and peripheral perfusion variables are shown in Figure 4. GEE analysis demonstrated a significant group-by-time interaction for dP/dtmax (*p* = 0.004). After Holm adjustment, the between-group difference remained statistically significant only at T8, with a higher dP/dtmax in the remimazolam group (estimated mean difference, 767.47 mmHg/s; 95% confidence interval, 394.41–1140.52 mmHg/s; Holm-adjusted *p* < 0.001).

No significant group-by-time interactions were observed for CI (*p* = 0.716) or CPI (*p* = 0.590), and no time-specific between-group contrasts for these variables remained statistically significant after Holm adjustment. In contrast, Pi showed a significant group-by-time interaction (*p* < 0.001), although none of the time-specific between-group contrasts remained significant after Holm adjustment. A post hoc complete-case sensitivity analysis for dP/dtmax yielded results consistent with the primary analysis.

### 3.6. Arterial Blood Gas Analysis

**Figure 5:**
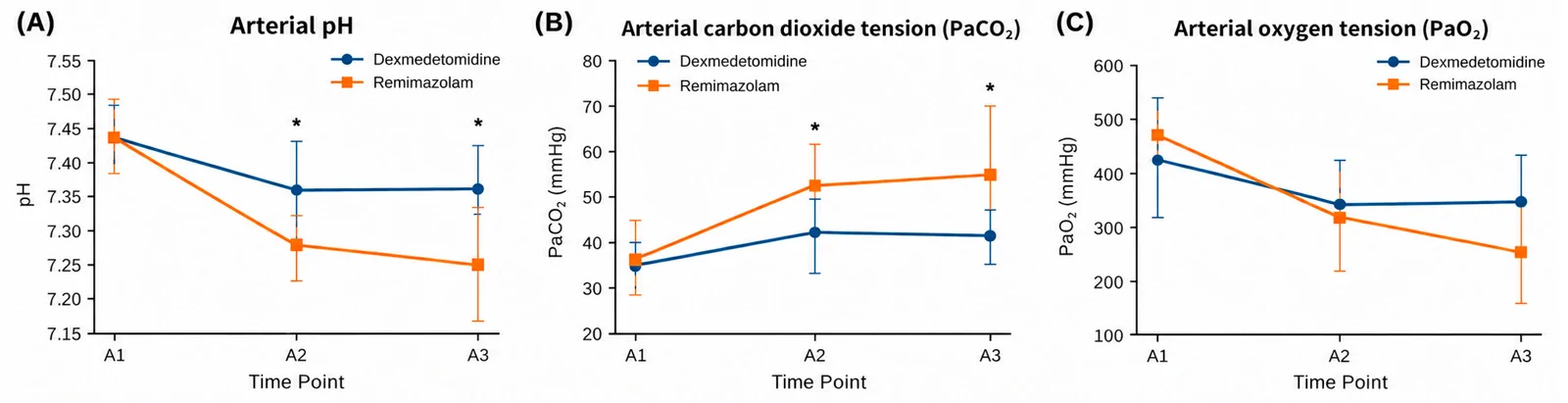
Serial changes in arterial blood gas variables during transfemoral transcatheter aortic valve implantation under monitored anesthesia care. (**A**) Arterial pH, (**B**) arterial carbon dioxide tension (PaCO_2_), and (**C**) arterial oxygen tension (PaO_2_). A1, immediately after arterial catheter placement and before initiation of sedation; A2, immediately before rapid ventricular pacing; A3, at completion of the procedure. Data are presented as mean ± SD. Longitudinal changes were analyzed using generalized estimating equations including treatment group, time, and the group-by-time interaction. *p*-values displayed in each panel represent the overall group-by-time interaction. *p*-values for time-specific between-group contrasts were adjusted for multiple comparisons within each outcome using the Holm method. An asterisk indicates a time-specific between-group difference that remained statistically significant after Holm adjustment (Holm-adjusted *p* < 0.05).

Serial changes in arterial blood gas variables are shown in Figure 5. GEE analysis demonstrated significant group-by-time interactions for arterial pH (*p* < 0.001) and PaCO_2_ (*p* < 0.001). After Holm adjustment, arterial pH was lower in the remimazolam group at A2 (estimated mean difference, −0.082; 95% confidence interval, −0.120 to −0.043; Holm-adjusted *p* < 0.001) and A3 (estimated mean difference, −0.107; 95% confidence interval, −0.158 to −0.056; Holm-adjusted *p* < 0.001). PaCO_2_ was higher in the remimazolam group at A2 (estimated mean difference, 11.14 mmHg; 95% confidence interval, 5.32–16.96 mmHg; Holm-adjusted *p* < 0.001) and A3 (estimated mean difference, 14.06 mmHg; 95% confidence interval, 6.28–21.85 mmHg; Holm-adjusted *p* < 0.001).

In contrast, no significant group-by-time interaction was observed for PaO_2_ (*p* = 0.099), and no time-specific between-group contrast remained statistically significant after Holm adjustment. No patient required escalation of airway support, including jaw thrust, airway adjunct placement, or bag-mask ventilation, and no patient required conversion to general anesthesia.

Among additional arterial blood gas-derived variables, significant group-by-time interactions were also observed for base excess (*p* = 0.010) and blood glucose (*p* = 0.005); however, none of their time-specific between-group contrasts remained significant after Holm adjustment. No significant group-by-time interactions were observed for HCO_3_^−^, hemoglobin, sodium, potassium, ionized calcium, chloride, anion gap, or lactate. Overall group-by-time interaction tests and detailed time-specific estimated mean differences with 95% confidence intervals and Holm-adjusted *p*-values are provided in Supplementary Tables S3 and S4, respectively.

## 4. Discussion

This prospective randomized trial compared remimazolam- and dexmedetomidine-based monitored anesthesia care during transfemoral TAVI. No statistically significant between-group differences were detected in the number of intraoperative hypotensive episodes or cumulative phenylephrine and norepinephrine doses, and longitudinal blood-pressure profiles did not differ significantly between the groups. Several secondary physiological variables showed significant differences in their temporal patterns; however, most time-specific between-group differences did not remain statistically significant after adjustment for multiple comparisons. The notable exceptions were a higher dP/dtmax in the remimazolam group at T8 and higher rSO_2_ values at selected time points. Remimazolam was also associated with higher PaCO_2_ and lower arterial pH at A2 and A3, while no significant group-by-time interaction was observed for PaO_2_. Clinically, remimazolam was associated with a shorter recovery time and fewer surgeon requests for deeper sedation, without the need for escalation to airway support or conversion to general anesthesia in either group. Taken together, these findings suggest differences in physiological response patterns and recovery characteristics between the two sedation strategies; however, the secondary physiological findings should be considered exploratory and should not be interpreted as evidence of overall clinical superiority of either regimen.

Several recent studies have compared remimazolam with dexmedetomidine during TAVI and other procedures performed under regional anesthesia or MAC. The major pharmacological and clinically relevant characteristics of remimazolam and dexmedetomidine in the context of procedural sedation are summarized in Table 3.

Kitaura et al. [20] reported similar hemodynamic profiles with remimazolam and dexmedetomidine, with faster postoperative recovery in the remimazolam group. Kim et al. [26] demonstrated non-inferiority of remimazolam with respect to recovery and perioperative safety in patients undergoing transfemoral TAVI under MAC, while Liang et al. [19] reported the feasibility of remimazolam-based sedation in this setting. Randomized trials and recent meta-analyses in other procedural settings have also suggested that remimazolam provides effective procedural sedation with favorable recovery characteristics compared with dexmedetomidine [22,23,24,25,26,29,30,31].

However, most previous studies focused primarily on procedural outcomes, recovery, and conventional hemodynamic measures. The present study extends this literature by evaluating the number of hypotensive episodes, cumulative phenylephrine and norepinephrine doses, and repeated changes in advanced hemodynamic, cerebral oxygenation, and arterial blood gas variables throughout the procedure. Specifically, pulse contour-derived cardiovascular variables, including dP/dtmax, CI, and CPI, were evaluated together with bilateral cerebral oxygen saturation and other physiological measurements. The principal findings of the present randomized trial are summarized in Table 4.

Regarding the primary hemodynamic outcomes, no statistically significant between-group differences were detected in the number of intraoperative hypotensive episodes, the proportion of patients experiencing at least one hypotensive episode, or cumulative phenylephrine and norepinephrine doses. Kim et al. [26] reported no significant difference in intraoperative vasopressor/inotrope use between remimazolam and dexmedetomidine during TAVI under monitored anesthesia care, although postoperative vasopressor/inotrope support was less frequent in the remimazolam group. Because the previous study evaluated the proportion of patients requiring vasopressor/inotrope support rather than the number of hypotensive episodes or cumulative vasopressor doses, direct comparison with the present findings is limited. These findings indicate that no between-group difference was detected in these intraoperative hemodynamic outcomes under the standardized management protocol used in this trial; however, they should not be interpreted as evidence of hemodynamic equivalence.

For conventional longitudinal hemodynamic measures, no significant group-by-time interactions were observed for MBP or invasive MAP. This pattern is broadly consistent with previous TAVI studies reporting no substantial between-group differences in conventional blood-pressure measures [19,20]. Heart rate showed a significant group-by-time interaction, indicating different temporal trajectories between the two groups; however, none of the time-specific between-group contrasts remained statistically significant after Holm adjustment. Thus, the conventional hemodynamic findings did not demonstrate a consistent time-specific advantage of either sedative regimen.

Pulse contour-derived dP/dtmax has been proposed as an index related to left ventricular systolic performance and may reflect changes in myocardial inotropic state [32]. However, because dP/dtmax is load dependent, its interpretation during TAVI is complicated by abrupt changes in preload, afterload, and arterial pressure waveforms associated with rapid ventricular pacing, balloon valvuloplasty, and valve deployment. A significant group-by-time interaction was observed for dP/dtmax, but after Holm adjustment, the between-group difference remained significant only at T8. In contrast, no significant group-by-time interactions were observed for CI or CPI. Therefore, the higher dP/dtmax at T8 with remimazolam should be regarded as an exploratory finding under specific hemodynamic conditions rather than evidence of superior overall cardiac performance.

SVV and PPV should be interpreted cautiously because their reliability may be limited during spontaneous breathing and rhythm irregularity, with rapid ventricular pacing during TAVI introducing additional hemodynamic variability. Although significant group-by-time interactions were observed for both SVV and PPV, no time-specific between-group contrasts remained statistically significant after Holm adjustment. These findings therefore represent exploratory temporal differences rather than definitive evidence of differential preload responsiveness between the two sedative regimens.

Another notable finding was the difference in the temporal pattern of cerebral oxygen saturation between the two groups. Significant group-by-time interactions were observed for both left and right rSO_2_, and after Holm adjustment, higher rSO_2_ values in the remimazolam group remained statistically significant at T5 and T7 on the left side and at T5 on the right side. Neurological complications remain important adverse events after TAVI [33,34]. Near-infrared spectroscopy provides a noninvasive method for continuous assessment of cerebral oxygenation during TAVI, and previous observational studies have reported associations between reductions in rSO_2_ and postoperative neurological complications, including delirium [35,36,37]. However, the higher rSO_2_ values with remimazolam occurred only at selected time points, and no statistically significant between-group difference was detected in clinically identified postoperative delirium. Therefore, these transient rSO_2_ differences should be regarded as exploratory physiological findings rather than evidence of cerebral protection or improved neurological outcomes.

Remimazolam was also associated with higher PaCO_2_ and lower arterial pH at A2 and A3, and hypercapnia-induced cerebral vasodilation may have increased cerebral blood flow and contributed, at least in part, to the observed rSO_2_ differences [38]. However, the present data do not establish a specific mechanism for these differences.

Procedural factors may also have influenced the secondary physiological measurements. Pre-balloon valvuloplasty was numerically more frequent in the dexmedetomidine group than in the remimazolam group (6/17 vs. 2/17), although the difference was not statistically significant. Because balloon valvuloplasty and associated rapid ventricular pacing can transiently alter arterial pressure, cardiac output, and cerebral perfusion, this imbalance may have introduced residual procedural confounding.

Remimazolam was associated with significantly higher PaCO_2_ and lower arterial pH than dexmedetomidine at A2 and A3, whereas no significant group-by-time interaction was observed for PaO_2_. These findings are compatible with greater carbon dioxide retention with remimazolam despite the absence of a detectable difference in the temporal pattern of arterial oxygenation. Although EtCO_2_ was continuously monitored as part of intraoperative respiratory surveillance, EtCO_2_ measurements during high-flow nasal oxygen may be affected by dilution of exhaled gas and variable sampling. Thus, continuous EtCO_2_ monitoring likely reduced the risk of unrecognized clinically important ventilatory deterioration, but it could not reliably exclude hypoventilation. The higher PaCO_2_ and lower arterial pH observed with remimazolam therefore provide more direct evidence of greater carbon dioxide retention despite maintained oxygenation.

The significantly shorter recovery time in the remimazolam group was another notable finding. Rapid recovery is a well-recognized pharmacological characteristic of remimazolam, attributable to its rapid metabolism by tissue esterases and limited accumulation [15,16,17,18]. Previous comparative studies and recent meta-analyses have also reported faster recovery with remimazolam than with dexmedetomidine across various procedural settings, including TAVI [20,22,23,24,25,26,29,30,31]. Our findings are consistent with this literature and extend these observations to a randomized TAVI population undergoing detailed longitudinal hemodynamic and physiological monitoring.

Surgeon requests for deeper sedation were markedly less frequent in the remimazolam group. Because surgeon requests represent a pragmatic clinical assessment rather than a validated sedation scale, this finding should be interpreted as a procedural outcome rather than an objective measure of sedation depth. In contrast, the longitudinal analysis of PSi showed no significant group-by-time interaction, and no time-specific between-group contrasts remained statistically significant after Holm adjustment. These findings suggest that surgeon-perceived adequacy of procedural sedation and a processed EEG-derived sedation index may reflect different aspects of sedation quality.

From a clinical perspective, the shorter recovery observed with remimazolam may be relevant to contemporary fast-track TAVI pathways, in which early neurological assessment, mobilization, and efficient postprocedural recovery are increasingly emphasized. However, the present study did not evaluate downstream patient-centered or resource-utilization outcomes, such as time to mobilization, intensive care utilization, or hospital length of stay. Larger multicenter randomized trials are needed to determine whether the observed differences in recovery characteristics and physiological responses translate into clinically meaningful outcomes.

This study has several limitations. First, this was a single-center trial with a relatively small sample size, limiting generalizability and statistical power. The sample-size calculation was based on an anticipated between-group difference in postoperative MAP rather than on the number of hypotensive episodes or cumulative vasopressor doses. Thus, the study was not specifically powered for these primary outcomes, and nonsignificant findings should not be interpreted as evidence of equivalence. Likewise, the multiple physiological endpoints were not individually powered and should be considered exploratory, particularly because multiplicity adjustment was performed within rather than across secondary outcomes.

Second, complete blinding was not feasible because the sedatives required different administration protocols. Although participants and surgeons were blinded and intraoperative management was standardized, anesthesia providers, outcome assessors, and data analysts were not blinded to group allocation, introducing potential performance, assessment, and analytical bias.

Third, continuous high-resolution arterial-pressure data were not retained, precluding assessment of hypotension duration or time-integrated burden. In addition, pulse-contour-derived dP/dtmax is load-dependent and should not be interpreted as a direct measure of intrinsic myocardial contractility, while SVV and PPV have limited reliability during spontaneous breathing, rhythm irregularity, and rapid ventricular pacing.

Fourth, surgeon requests for deeper sedation reflected clinical assessment of apparent wakefulness or patient movement and therefore represent a pragmatic rather than validated measure of sedation depth. Postoperative delirium was likewise identified clinically rather than using a validated instrument such as the Confusion Assessment Method or the Confusion Assessment Method for the Intensive Care Unit. Although sedation depth was monitored using PSi, raw EEG waveforms and detailed electrophysiological measures were not collected, precluding direct evaluation of their relationship with cerebral oxygenation.

Finally, although EtCO_2_ was continuously monitored, its reliability may have been limited during high-flow nasal oxygen because of dilution of exhaled gas and variable sampling. In addition, respiratory events such as apnea not requiring intervention, bradypnea, and transient upper-airway obstruction were not prospectively collected as separate predefined outcomes.

## 5. Conclusions

In patients undergoing transfemoral TAVI under monitored anesthesia care, no statistically significant between-group differences were detected in the number of intraoperative hypotensive episodes or cumulative phenylephrine and norepinephrine doses, and no significant group-by-time interactions were observed for longitudinal blood-pressure measures. However, the trial was not designed or powered to establish equivalence with respect to these outcomes or overall hemodynamic stability. Remimazolam was associated with a shorter recovery time and fewer surgeon requests for deeper sedation. Selected differences in cerebral oxygenation and other secondary physiological variables were observed, and remimazolam was associated with higher PaCO_2_ and lower arterial pH at specific procedural time points; these findings should be considered exploratory. Overall, remimazolam appears to be a feasible alternative to dexmedetomidine for monitored anesthesia care during transfemoral TAVI. Larger multicenter randomized studies are needed to determine whether the observed differences in recovery and physiological responses translate into clinically meaningful patient-centered outcomes.

## Figures and Tables

**Table 1:** Baseline and Perioperative Characteristics of Patients Undergoing Monitored Anesthesia Care for Transfemoral Transcatheter Aortic Valve Implantation.

Variable	Dexmedetomidine (*n* = 17)	Remimazolam (*n* = 17)	*p* Value
**Demographics and clinical characteristics**			
Age, years	83.4 ± 3.2	83.0 ± 3.7	0.767
Height, cm	152.1 ± 8.4	157.4 ± 8.0	0.070
Weight, kg	53.1 ± 7.9	57.1 ± 9.6	0.204
Male sex, *n* (%)	5 (29.4)	7 (41.2)	0.721
Coronary artery disease, *n* (%)	4 (23.5)	9 (52.9)	0.157
Pulmonary hypertension, *n* (%)	4 (23.5)	3 (17.6)	1.000
Hypertension, *n* (%)	15 (88.2)	12 (70.6)	0.398
Diabetes mellitus, *n* (%)	4 (23.5)	8 (47.1)	0.282
Left ventricular ejection fraction, %	58.6 ± 5.5	59.1 ± 9.1	0.228
Aortic valve area, cm^2^	0.7 ± 0.2	0.8 ± 0.2	0.051
**Procedural and anesthetic characteristics**			
Total dexmedetomidine dose, μg	172.2 ± 40.3	-	-
Total remimazolam dose, mg	-	28.6 ± 9.7	-
Procedure time, min	94.4 ± 21.6	94.4 ± 37.8	0.604
Anesthesia time, min	127.8 ± 24.2	129.1 ± 38.3	0.822

Data are presented as mean ± SD or *n* (%). Continuous variables were compared using Welch’s *t*-test or the Mann–Whitney U test, as appropriate. Categorical variables were compared using Fisher’s exact test. Total study-sedative doses are presented descriptively because dexmedetomidine and remimazolam were administered using different dosing regimens and units. Bold text indicates category headings.

**Table 2:** Primary and Perioperative Outcomes.

Variable	Dexmedetomidine (*n* = 17)	Remimazolam (*n* = 17)	*p* Value
**Primary Outcomes**			
Number of intraoperative hypotensive episodes	3.1 ± 3.7	2.1 ± 2.9	0.477
Cumulative phenylephrine dose, μg	263.8 ± 376.1	161.2 ± 208.3	0.512
Cumulative norepinephrine dose, μg	333.4 ± 663.7	302.9 ± 695.7	0.762
**Other Perioperative Outcomes**			
Patients with ≥1 hypotensive episode, *n* (%)	12 (70.6)	9 (52.9)	0.481
Recovery time, min	32.4 ± 12.5	13.5 ± 7.2	<0.001
Patients with a surgeon request for deeper sedation, *n* (%)	13 (76.5)	2 (11.8)	<0.001
Cumulative remifentanil dose, μg	245.8 ± 54.6	245.0 ± 85.7	0.992
**Procedural Outcomes**			
Valve recapture, *n* (%)	3 (17.6)	2 (11.8)	1.000
Pre-balloon valvuloplasty, *n* (%)	6 (35.3)	2 (11.8)	0.225
Carotid shielding, *n* (%)	11 (64.7)	11 (64.7)	1.000
**Postprocedural Outcomes**			
Atrioventricular block, *n* (%)	7 (41.2)	6 (35.3)	1.000
Clinically identified postoperative delirium, *n* (%)	2 (11.8)	2 (11.8)	1.000

Data are presented as mean ± SD or *n* (%). Continuous variables were compared using Welch’s *t*-test or the Mann–Whitney U test, as appropriate. Categorical variables were compared using Fisher’s exact test. Bold text indicates category headings.

**Table 3:** Comparison of Remimazolam and Dexmedetomidine for Procedural Sedation.

Characteristic	Remimazolam	Dexmedetomidine
Pharmacologic class	Ultrashort-acting benzodiazepine [15,16]	Highly selective α_2_-adrenergic receptor agonist [11,12]
Sedation characteristics	Provides effective procedural sedation; rapid titration and recovery have been reported across procedural settings [17,18,19,20,21]	Provides cooperative sedation and generally preserves spontaneous ventilation [11,12]
Metabolism and recovery	Rapidly metabolized by tissue esterases to an inactive metabolite, resulting in rapid onset, predictable recovery, and limited accumulation [15,16]	Several comparative studies have reported slower recovery than with remimazolam [20,22,23,24,25,26,29,30,31]
Respiratory considerations	Limited respiratory depression has been reported in procedural sedation studies, although clinically relevant ventilatory depression may occur and requires appropriate monitoring [17,18,19,20,21]	Respiratory depression is generally limited, with relative preservation of spontaneous ventilation [11,12]
Hemodynamic considerations	Relatively stable hemodynamics have been reported [17,18,19,20,21]; comparative studies have suggested a lower incidence of bradycardia than with dexmedetomidine, whereas findings for hypotension have been less consistent [22,23,24,25]	Dose-dependent bradycardia and hypotension may occur; transient hypertension can occur during loading [13,14]
Evidence in TAVI	Clinical studies have reported feasibility and favorable recovery characteristics; one propensity score-matched study demonstrated non-inferiority for recovery and perioperative safety [19,20,26]	Commonly used comparator for MAC in TAVI and other procedural settings [20,26]
Key practical consideration for MAC	Rapid recovery and limited accumulation may facilitate early postprocedural assessment, while ventilation should remain closely monitored	Cooperative sedation with limited respiratory depression may be advantageous, while bradycardia and hypotension require close hemodynamic monitoring

**Notes:** This table summarizes pharmacological and clinical characteristics reported in the cited literature and is not intended to imply superiority of either sedative. Findings from the present randomized trial are reported in detail in Section 3. **Abbreviations**: MAC, monitored anesthesia care; TAVI, transcatheter aortic valve implantation.

**Table 4:** Summary of Principal Findings of the Present Randomized Trial.

Outcome	Dexmedetomidine	Remimazolam	Between-Group Finding/Interpretation
Intraoperative hypotensive episodes	3.1 ± 3.7 episodes; ≥1 episode: 12/17 (70.6%)	2.1 ± 2.9 episodes; ≥1 episode: 9/17 (52.9%)	Episode count *p* = 0.477; ≥1 episode *p* = 0.481.
Vasopressor doses	Phenylephrine 263.8 ± 376.1 μg; norepinephrine 333.4 ± 663.7 μg	Phenylephrine 161.2 ± 208.3 μg; norepinephrine 302.9 ± 695.7 μg	No significant difference detected (phenylephrine *p* = 0.512; norepinephrine *p* = 0.762).
Longitudinal blood pressure	—	—	No significant group-by-time interaction for MBP (*p* = 0.197) or invasive MAP (*p* = 0.160).
Heart rate	—	—	Significant group-by-time interaction (*p* < 0.001); no time-specific contrast remained significant after Holm adjustment.
Cardiac performance	—	—	dP/dtmax: interaction *p* = 0.004; only T8 remained significant after Holm adjustment (remimazolam − dexmedetomidine, 767.47 mmHg/s; 95% confidence interval, 394.41–1140.52 mmHg/s; Holm-adjusted *p* < 0.001). No significant interaction for CI (*p* = 0.716) or CPI (*p* = 0.590).
Cerebral oxygenation (rSO_2_)	—	—	Both hemisphere-specific interactions remained significant after Holm adjustment (Holm-adjusted *p* = 0.012 for each hemisphere); higher values with remimazolam at left T5/T7 and right T5 only.
Arterial blood gas/ventilation	Lower PaCO_2_ and higher pH at A2/A3 relative to remimazolam	Higher PaCO_2_ and lower pH at A2/A3	pH and PaCO_2_ interactions both *p* < 0.001; PaO_2_ interaction *p* = 0.099. Findings are compatible with greater carbon dioxide retention with remimazolam, without a significant between-group difference in the temporal pattern of PaO_2_.
Sedation depth (PSi)	—	—	No significant group-by-time interaction (*p* = 0.401) or Holm-adjusted time-specific difference.
Surgeon requests for deeper sedation	13/17 (76.5%)	2/17 (11.8%)	Less frequent with remimazolam (*p* < 0.001); a pragmatic procedural outcome, not a validated measure of sedation depth.
Recovery time	32.4 ± 12.5 min	13.5 ± 7.2 min	Shorter with remimazolam (*p* < 0.001).
Airway support/conversion to general anesthesia	None	None	No jaw thrust, airway adjunct placement, bag-mask ventilation, or conversion to general anesthesia.
Clinically identified postoperative delirium	2/17 (11.8%)	2/17 (11.8%)	No significant difference detected (*p* = 1.000); assessment was clinical rather than validated.

**Notes:** Data are mean ± SD or *n* (%). Longitudinal outcomes were analyzed by GEE with group, time, and group-by-time interaction; *p* values for time-specific contrasts were adjusted using the Holm method. This table summarizes principal findings only; complete longitudinal results are provided in Supplementary Tables S1–S4. Secondary physiological findings are exploratory and do not establish superiority of either regimen with respect to cardiovascular performance or cerebral oxygenation. **Abbreviations**: CI, cardiac index; CPI, cardiac power index; dP/dtmax, maximum rate of arterial pressure rise derived from pulse-contour analysis; GEE, generalized estimating equations; MAP, mean arterial pressure; MBP, mean blood pressure; PaCO_2_, arterial carbon dioxide tension; PaO_2_, arterial oxygen tension; PSi, Patient State Index; rSO_2_, regional cerebral oxygen saturation.

## Data Availability

The data presented in this study are available from the corresponding author upon reasonable request. The data are not publicly available because of institutional and participant privacy restrictions.

## Supplementary Materials

The following supporting information can be downloaded at: https://www.mdpi.com/article/10.3390/jcm15187182/s1, Table S1: Global Group-by-Time Interaction Tests From GEE Analyses of Longitudinal Hemodynamic and Physiological Outcomes; Table S2: Time-Specific Between-Group Contrasts From GEE Analyses of Longitudinal Hemodynamic and Physiological Outcomes; Table S3: Global Group-by-Time Interaction Tests From GEE Analyses of Arterial Blood Gas and Laboratory Outcomes; Table S4: Time-Specific Between-Group Contrasts From GEE Analyses of Arterial Blood Gas and Laboratory Outcomes.

## Abbreviations

The following abbreviations are used in this manuscript:

TAVI	transcatheter aortic valve implantation
tf-TAVI	transfemoral transcatheter aortic valve implantation
MAC	monitored anesthesia care
HR	heart rate
MBP	mean blood pressure
MAP	mean arterial pressure
SpO_2_	peripheral oxygen saturation
EtCO_2_	end-tidal carbon dioxide
PaCO_2_	arterial carbon dioxide tension
PaO_2_	arterial oxygen tension
PVI	pleth variability index
Pi	perfusion index
SpHb	noninvasive hemoglobin concentration
ORi	oxygen reserve index
PSi	Patient State Index
rSO_2_	regional cerebral oxygen saturation
CI	cardiac index
SVI	stroke volume index
SVV	stroke volume variation
PPV	pulse pressure variation
SVRI	systemic vascular resistance index
CPI	cardiac power index
dP/dtmax	maximum rate of arterial pressure rise derived from pulse-contour analysis
RSS	Ramsay Sedation Scale
EEG	electroencephalography
GEE	generalized estimating equations
SD	standard deviation
