# Supplementary material for: Remimazolam Versus Dexmedetomidine for Monitored Anesthesia Care in Patients Undergoing Transfemoral Transcatheter Aortic Valve Implantation: A Randomized Clinical Trial
Source: J Clin Med. 2026 Sep 16;15(18):7182. doi: 10.3390/jcm15187182 (PMC13607414; doi:10.3390/jcm15187182)
Supplement: Supplementary file 1 [file jcm-15-07182-s001.zip › Supplementary_Table_S1_JCM.pdf]

**Supplementary Table S1. Global Group-by-Time Interaction Tests From GEE Analyses of Longitudinal Hemodynamic and Physiological Outcomes**

| Outcome | Time points | df | Wald $\chi^2$ for Group×Time | P value |
|---|---|---|---|---|
| ***Hemodynamic variables*** | | | | |
| MBP | T1–T9 | 8 | 11.08 | 0.197 |
| MAP | T1–T8 | 7 | 10.50 | 0.160 |
| HR | T1–T9 | 8 | 39.50 | <0.001 |
| $SpO_2$ | T1–T9 | 8 | 10.37 | 0.240 |
| SVI | T1–T8 | 7 | 25.10 | <0.001 |
| SVV | T1–T8 | 7 | 18.62 | 0.009 |
| PPV | T1–T8 | 7 | 15.9 | 0.026 |
| SVRI | T1–T8 | 7 | 11.70 | 0.110 |
| ***Cardiovascular performance*** | | | | |
| CI | T1–T8 | 7 | 4.54 | 0.716 |
| CPI | T1–T8 | 7 | 5.58 | 0.590 |
| dP/dtmax | T1–T8 | 7 | 20.70 | 0.004 |
| ***Sedation and peripheral physiological variables*** | | | | |
| PSi | T1–T8 | 7 | 7.28 | 0.401 |
| PVI | T1–T8 | 7 | 22.3 | 0.002 |
| Pi | T1–T8 | 7 | 39.07 | <0.001 |
| ORi | T1–T8 | 7 | 18.27 | 0.011 |
| SpHb | T1–T8 | 7 | 21.6 | 0.003 |
| ***Cerebral oxygenation*** | | | | |
| Left $rSO_2$ | T1–T8 | 7 | 19.90 | 0.012* |
| Right $rSO_2$ | T1–T8 | 7 | 19.30 | 0.012* |

**Notes:** P values represent joint Wald tests of the group-by-time interaction from generalized estimating equation models. P values are unadjusted unless otherwise indicated. *Holm-adjusted across the two hemisphere-specific interaction tests for bilateral $rSO_2$.

**Abbreviations:** MBP, mean blood pressure; MAP, invasive mean arterial pressure; HR, heart rate; $SpO_2$, peripheral oxygen saturation; SVI, stroke volume index; SVV, stroke volume variation; PPV, pulse pressure variation; SVRI, systemic vascular resistance index; CI, cardiac index; CPI, cardiac power index; dP/dtmax, maximum rate of arterial pressure rise derived from pulse-contour analysis; PSi, Patient State Index; PVI, pleth variability index; Pi, perfusion index; ORi, oxygen reserve index; SpHb, noninvasive hemoglobin concentration; $rSO_2$, regional cerebral oxygen saturation.
