# Supplementary material for: Remimazolam Versus Dexmedetomidine for Monitored Anesthesia Care in Patients Undergoing Transfemoral Transcatheter Aortic Valve Implantation: A Randomized Clinical Trial
Source: J Clin Med. 2026 Sep 16;15(18):7182. doi: 10.3390/jcm15187182 (PMC13607414; doi:10.3390/jcm15187182)
Supplement: Supplementary file 1 [file jcm-15-07182-s001.zip › Supplementary_Table_S2_JCM.pdf]

Supplementary Table S2. Time-Specific Between-Group Contrasts From GEE Analyses of Longitudinal Hemodynamic and Physiological Outcomes

Outcome	Time point	Estimated mean difference (Remimazolam − Dexmedetomidine)	95% confidence interval	Raw P value	Holm-adjusted P value
		Hemodynamic variables			
**MBP**	T1	-1.24	-11.67 to 9.20	0.817	1.000
	T2	-1.41	-11.61 to 8.79	0.786	1.000
	T3	-6.94	-18.16 to 4.28	0.226	1.000
	T4	-7.35	-17.01 to 2.31	0.136	0.815
	T5	-9.18	-16.41 to -1.94	0.013	0.116
	T6	-13.18	-25.80 to -0.55	0.041	0.285
	T7	-11.00	-21.04 to -0.96	0.032	0.254
	T8	0.18	-9.98 to 10.33	0.973	1.000
	T9	3.18	-7.70 to 14.06	0.567	1.000
**MAP**	T1	-5.35	-16.10 to 5.43	0.331	0.992
	T2	-4.65	-14.60 to 5.35	0.362	0.992
	T3	-6.82	-18.30 to 4.67	0.245	0.978
	T4	-7.76	-17.30 to 1.73	0.109	0.544
	T5	-9.65	-17.70 to -1.60	0.019	0.140
	T6	-17.76	-32.40 to -3.10	0.018	0.140
	T7	-9.88	-19.90 to 0.12	0.053	0.316
	T8	2.47	-8.20 to 13.16	0.651	0.992
**HR**	T1	-3.94	-13.96 to 6.08	0.441	1.000
	T2	0.35	-8.39 to 9.09	0.937	1.000
	T3	6.47	-0.86 to 13.80	0.083	0.417
	T4	10.82	1.33 to 20.31	0.025	0.152
	T5	10.24	1.90 to 18.57	0.016	0.113
	T6	1.24	-9.20 to 11.67	0.817	1.000
	T7	4.71	-6.14 to 15.55	0.395	1.000
	T8	12.24	2.89 to 21.58	0.010	0.093
	T9	13.94	3.16 to 24.72	0.011	0.093
**SpO_2**	T1	0.29	-0.27 to 0.85	0.303	1.000
	T2	0.47	-0.31 to 1.25	0.239	1.000
	T3	-0.18	-0.51 to 0.16	0.303	1.000
	T4	0.29	-0.27 to 0.85	0.303	1.000
	T5	0.00	NE	NE	NE
	T6	-0.06	-0.58 to 0.46	0.826	1.000
	T7	0.00	-0.57 to 0.57	1.000	1.000
	T8	-0.12	-0.50 to 0.26	0.543	1.000
	T9	0.71	-1.02 to 2.43	0.422	1.000
**SVI**	T1	1.29	-5.74 to 8.33	0.718	1.000
	T2	0.64	-7.35 to 8.63	0.876	1.000
	T3	-3.97	-10.61 to 2.66	0.241	1.000
	T4	-5.35	-10.21 to -0.48	0.031	0.250
	T5	-5.56	-13.93 to 2.80	0.192	1.000
	T6	5.01	-1.63 to 11.65	0.139	0.973
	T7	5.17	-3.35 to 13.70	0.234	1.000
	T8	1.30	-5.97 to 8.58	0.725	1.000
**SVV**	T1	-0.36	-7.23 to 6.50	0.917	1.000

Outcome	Time point	Estimated mean difference (Remimazolam − Dexmedetomidine)	95% confidence interval	Raw P value	Holm-adjusted P value
	T2	2.74	-4.11 to 9.59	0.433	1.000
	T3	2.54	-4.07 to 9.16	0.452	1.000
	T4	6.35	-0.56 to 13.26	0.072	0.503
	T5	6.88	1.20 to 12.56	0.018	0.141
	T6	2.28	-2.46 to 7.02	0.345	1.000
	T7	2.51	-2.76 to 7.79	0.351	1.000
	T8	3.99	-0.96 to 8.93	0.114	0.683
**PPV**	T1	-0.31	-5.17 to 4.55	0.901	1.000
	T2	-1.42	-6.16 to 3.33	0.559	1.000
	T3	1.07	-3.93 to 6.08	0.674	1.000
	T4	5.47	0.34 to 10.61	0.037	0.256
	T5	4.98	0.43 to 9.53	0.032	0.255
	T6	1.23	-4.00 to 6.46	0.645	1.000
	T7	-1.18	-6.84 to 4.49	0.684	1.000
	T8	1.07	-4.91 to 7.04	0.726	1.000
**SVRI**	T1	-341	-836 to 154	0.177	0.708
	T2	-312	-769 to 144	0.180	0.708
	T3	-295	-823 to 234	0.275	0.708
	T4	-265	-609 to 79	0.131	0.656
	T5	-429	-837 to -20	0.040	0.237
	T6	-720	-1130 to -310	<0.001	0.005
	T7	-714	-1203 to -224	0.004	0.030
	T8	-390	-993 to 212	0.204	0.708
		Cardiovascular performance			
**CI**	T1	0.13	-0.31 to 0.57	0.565	1.000
	T2	0.17	-0.30 to 0.64	0.481	1.000
	T3	0.03	-0.40 to 0.45	0.909	1.000
	T4	0.04	-0.34 to 0.41	0.837	1.000
	T5	0.13	-0.51 to 0.76	0.691	1.000
	T6	0.30	-0.26 to 0.85	0.293	1.000
	T7	0.51	-0.18 to 1.20	0.145	1.000
	T8	0.42	-0.17 to 1.01	0.161	1.000
**CPI**	T1	-0.005	-0.130 to 0.120	0.941	1.000
	T2	0.002	-0.135 to 0.139	0.980	1.000
	T3	-0.049	-0.178 to 0.079	0.452	1.000
	T4	-0.035	-0.145 to 0.075	0.530	1.000
	T5	-0.037	-0.162 to 0.088	0.560	1.000
	T6	-0.091	-0.287 to 0.106	0.367	1.000
	T7	0.029	-0.090 to 0.148	0.635	1.000
	T8	0.088	-0.033 to 0.210	0.155	1.000
**dP/dtmax**	T1	271.18	-17.65 to 560.00	0.066	0.361
	T2	302.09	-13.02 to 617.20	0.060	0.361
	T3	201.09	-88.60 to 490.79	0.174	0.695
	T4	100.09	-145.01 to 345.20	0.423	0.913
	T5	105.47	-95.80 to 306.74	0.304	0.913
	T6	-180.78	-762.84 to 401.27	0.543	0.913
	T7	387.97	58.87 to 717.06	0.021	0.146

Outcome	Time point	Estimated mean difference (Remimazolam − Dexmedetomidine)	95% confidence interval	Raw P value	Holm-adjusted P value
	T8	767.47	394.41 to 1140.52	<0.001	<0.001
		Sedation and peripheral physiological variables			
**PSi**	T1	1.71	-0.45 to 3.86	0.121	0.581
	T2	0.71	-11.26 to 12.67	0.908	0.908
	T3	13.29	-1.00 to 27.59	0.068	0.410
	T4	14.35	0.40 to 28.30	0.044	0.350
	T5	12.88	0.20 to 25.56	0.046	0.350
	T6	9.65	-5.29 to 24.59	0.206	0.617
	T7	6.76	-7.35 to 20.88	0.348	0.695
	T8	12.88	-3.19 to 28.96	0.116	0.581
**PVI**	T1	-1.88	-10.43 to 6.66	0.666	1.000
	T2	-2.06	-10.73 to 6.61	0.641	1.000
	T3	2.35	-6.63 to 11.34	0.608	1.000
	T4	2.41	-7.13 to 11.96	0.620	1.000
	T5	5.82	-1.73 to 13.37	0.131	0.907
	T6	-6.00	-13.76 to 1.76	0.130	0.907
	T7	-7.29	-15.85 to 1.26	0.095	0.757
	T8	1.76	-7.37 to 10.90	0.705	1.000
**Pi**	T1	0.19	-0.84 to 1.21	0.720	1.000
	T2	-0.19	-1.28 to 0.90	0.731	1.000
	T3	-0.14	-1.47 to 1.19	0.835	1.000
	T4	0.47	-0.87 to 1.81	0.494	1.000
	T5	0.31	-0.86 to 1.48	0.600	1.000
	T6	0.07	-1.25 to 1.38	0.918	1.000
	T7	-0.00	-1.07 to 1.06	0.997	1.000
	T8	-0.99	-1.97 to -0.00	0.049	0.394
**ORi**	T1	0.095	-0.044 to 0.235	0.180	0.901
	T2	0.041	-0.095 to 0.177	0.553	1.000
	T3	-0.113	-0.254 to 0.028	0.116	0.731
	T4	-0.105	-0.232 to 0.022	0.104	0.731
	T5	-0.105	-0.263 to 0.053	0.191	0.901
	T6	-0.038	-0.251 to 0.174	0.724	1.000
	T7	-0.035	-0.224 to 0.153	0.713	1.000
	T8	-0.214	-0.421 to -0.006	0.044	0.351
**SpHb**	T1	0.31	-0.44 to 1.05	0.419	1.000
	T2	0.52	-0.26 to 1.29	0.192	1.000
	T3	0.45	-0.36 to 1.26	0.280	1.000
	T4	0.58	-0.23 to 1.38	0.159	1.000
	T5	0.55	-0.22 to 1.31	0.163	1.000
	T6	0.29	-0.51 to 1.10	0.475	1.000
	T7	0.48	-0.31 to 1.26	0.233	1.000
	T8	0.35	-0.44 to 1.14	0.388	1.000
		Cerebral oxygenation			
**Left rSO_2**	T1	1.82	-1.98 to 5.63	0.347	1.000
	T2	1.24	-3.02 to 5.49	0.569	1.000
	T3	2.65	-1.21 to 6.51	0.179	0.895
	T4	4.65	0.75 to 8.54	0.019	0.194

Outcome	Time point	Estimated mean difference (Remimazolam − Dexmedetomidine)	95% confidence interval	Raw P value	Holm-adjusted P value
	T5	6.00	2.40 to 9.60	0.001	0.016
	T6	5.65	1.08 to 10.22	0.015	0.170
	T7	7.59	3.29 to 11.89	<0.001	0.009
	T8	6.06	0.94 to 11.18	0.020	0.194
**Right rSO_2**	T1	-0.47	-4.29 to 3.35	0.809	1.000
	T2	1.41	-2.57 to 5.39	0.487	1.000
	T3	2.47	-0.81 to 5.76	0.141	0.843
	T4	3.59	-0.01 to 7.18	0.050	0.404
	T5	5.59	2.22 to 8.95	0.001	0.016
	T6	5.76	1.45 to 10.08	0.009	0.105
	T7	6.12	1.75 to 10.49	0.006	0.079
	T8	4.76	-0.34 to 9.87	0.067	0.472

Notes: Differences are expressed as remimazolam minus dexmedetomidine. The 95% confidence intervals are pointwise. Holm-adjusted P values control multiplicity across time points within each outcome; bilateral rSO_2 was treated as one 16-contrast family. Time-specific significance was based on adjusted P values. Contrasts from outcomes with nonsignificant global interactions are shown for completeness and interpreted cautiously. At T5, SpO_2 was 100% in both groups, so the confidence interval and P value were not estimable (NE).

Abbreviations: as defined in Supplementary Table S1; NE, not estimable.
