# Supplementary material for: Remimazolam Versus Dexmedetomidine for Monitored Anesthesia Care in Patients Undergoing Transfemoral Transcatheter Aortic Valve Implantation: A Randomized Clinical Trial
Source: J Clin Med. 2026 Sep 16;15(18):7182. doi: 10.3390/jcm15187182 (PMC13607414; doi:10.3390/jcm15187182)
Supplement: Supplementary file 1 [file jcm-15-07182-s001.zip › Supplementary_Table_S3_JCM.pdf]

Supplementary Table S3. Global Group-by-Time Interaction Tests From GEE Analyses of Arterial Blood Gas and Laboratory Outcomes

Outcome	Time points	df	Wald χ^2 for Group×Time	P value
pH	A1–A3	2	31.69	<0.001
$PaCO_2$	A1–A3	2	24.94	<0.001
PaO_2	A1–A3	2	4.62	0.099
HCO_3^-	A1–A3	2	0.83	0.661
Base excess	A1–A3	2	9.30	0.010
Hemoglobin	A1–A3	2	0.93	0.627
Sodium	A1–A3	2	1.48	0.477
Potassium	A1–A3	2	1.70	0.428
Ionized calcium	A1–A3	2	4.34	0.114
Chloride	A1–A3	2	2.23	0.328
Anion gap	A1–A3	2	1.17	0.558
Glucose	A1–A3	2	10.67	0.005
Lactate	A1–A3	2	0.60	0.740

Notes: P values represent joint Wald tests of the group-by-time interaction from generalized estimating equation models. P values are unadjusted across distinct outcomes. A1, immediately after arterial catheter placement and before initiation of sedation; A2, immediately before rapid ventricular pacing; A3, at completion of the procedure.

Abbreviations: GEE, generalized estimating equation; $PaCO_2$, arterial carbon dioxide tension; PaO_2, arterial oxygen tension; HCO_3^-, bicarbonate.
