# Supplementary material for: Remimazolam Versus Dexmedetomidine for Monitored Anesthesia Care in Patients Undergoing Transfemoral Transcatheter Aortic Valve Implantation: A Randomized Clinical Trial
Source: J Clin Med. 2026 Sep 16;15(18):7182. doi: 10.3390/jcm15187182 (PMC13607414; doi:10.3390/jcm15187182)
Supplement: Supplementary file 1 [file jcm-15-07182-s001.zip › Supplementary_Table_S4_JCM.pdf]

Supplementary Table S4. Time-Specific Between-Group Contrasts From GEE Analyses of Arterial Blood Gas and Laboratory Outcomes

Outcome	Time point	Estimated mean difference (Remimazolam − Dexmedetomidine)	95% confidence interval	Raw P value	Holm-adjusted P value
pH	A1	0.001	−0.030 to 0.032	0.946	0.946
pH	A2	−0.082	−0.120 to −0.043	<0.001	<0.001
pH	A3	−0.107	−0.158 to −0.056	<0.001	<0.001
$PaCO_2$	A1	1.08	−3.05 to 5.21	0.608	0.608
$PaCO_2$	A2	11.14	5.32 to 16.96	<0.001	<0.001
$PaCO_2$	A3	14.06	6.28 to 21.85	<0.001	<0.001
PaO_2	A1	44.14	−15.22 to 103.50	0.145	0.290
PaO_2	A2	−40.01	−115.82 to 35.80	0.301	0.301
PaO_2	A3	−65.04	−136.28 to 6.21	0.074	0.221
HCO_3^-	A1	0.43	−1.68 to 2.54	0.690	1.000
HCO_3^-	A2	1.01	−1.16 to 3.18	0.360	1.000
HCO_3^-	A3	0.51	−1.29 to 2.30	0.581	1.000
Base excess	A1	0.41	−1.61 to 2.42	0.693	0.908
Base excess	A2	−0.78	−2.83 to 1.27	0.454	0.908
Base excess	A3	−1.69	−3.63 to 0.25	0.088	0.264
Hemoglobin	A1	0.71	−0.10 to 1.52	0.085	0.254
Hemoglobin	A2	0.65	−0.13 to 1.42	0.103	0.254
Hemoglobin	A3	1.46	−0.34 to 3.26	0.113	0.254
Sodium	A1	−0.48	−2.25 to 1.30	0.598	1.000
Sodium	A2	−0.19	−1.89 to 1.52	0.829	1.000
Sodium	A3	0.01	−1.81 to 1.82	0.995	1.000
Potassium	A1	0.16	−0.12 to 0.45	0.268	0.804
Potassium	A2	0.17	−0.15 to 0.49	0.292	0.804
Potassium	A3	0.06	−0.27 to 0.38	0.741	0.804
Ionized calcium	A1	0.02	−0.01 to 0.04	0.238	0.476
Ionized calcium	A2	0.03	−0.01 to 0.07	0.124	0.372
Ionized calcium	A3	0.00	−0.03 to 0.04	0.947	0.947
Chloride	A1	−0.47	−2.83 to 1.89	0.696	1.000
Chloride	A2	−0.71	−3.45 to 2.04	0.614	1.000
Chloride	A3	0.24	−2.39 to 2.86	0.861	1.000
Anion gap	A1	−0.26	−2.07 to 1.54	0.774	1.000
Anion gap	A2	−0.33	−2.12 to 1.46	0.719	1.000
Anion gap	A3	−0.90	−2.55 to 0.75	0.285	0.855
Glucose	A1	−2.59	−27.44 to 22.26	0.838	0.838
Glucose	A2	−25.00	−49.32 to −0.68	0.044	0.132
Glucose	A3	−25.71	−50.90 to −0.51	0.046	0.132
Lactate	A1	−0.08	−0.47 to 0.32	0.701	0.701
Lactate	A2	−0.16	−0.45 to 0.13	0.273	0.689

Outcome	Time point	Estimated mean difference (Remimazolam − Dexmedetomidine)	95% confidence interval	Raw P value	Holm-adjusted P value
Lactate	A3	−0.15	−0.41 to 0.10	0.230	0.689

Notes: Differences are expressed as remimazolam minus dexmedetomidine. The 95% confidence intervals are pointwise and were not adjusted for multiplicity. Time-specific P values were adjusted across A1–A3 within each outcome using the Holm method, and statistical significance for time-specific contrasts was based on the Holm-adjusted P values. Contrasts from outcomes with nonsignificant global group-by-time interactions are shown for completeness and should be interpreted cautiously. A1, immediately after arterial catheter placement and before initiation of sedation; A2, immediately before rapid ventricular pacing; A3, at completion of the procedure.

Abbreviations: $PaCO_2$, arterial carbon dioxide tension; PaO_2, arterial oxygen tension; HCO_3^-, bicarbonate.
